# Viability testing to guide myocardial revascularisation in patients with heart failure

**DOI:** 10.1007/s12055-017-0637-4

**Published:** 2018-02-17

**Authors:** Thomas J. Cahill, Rajesh K. Kharbanda

**Affiliations:** Oxford Heart Centre, Oxford University Hospitals NHS Foundation Trust, Headley Way, Oxford, OX3 9DU UK

**Keywords:** Coronary artery disease, Heart failure, Ischaemic cardiomyopathy

## Abstract

Myocardial revascularisation has the potential to restore ventricular function and improve survival in patients with heart failure due to underlying coronary artery disease. Viability testing is routinely used to identify which patients are likely to benefit, given that revascularisation may entail substantial procedural risk. However, while the concept of viability testing and revascularisation of patients with ‘hibernating myocardium’ is strongly supported by observational series, randomised studies have failed to demonstrate clear benefit. This divergence in the evidence base is reflected in current European and US guidelines, in which viability testing has a class II recommendation. In this article, we review the current evidence for routine viability testing prior to revascularisation of patients with heart failure, outline its use in clinical practice and discuss ongoing trials in the field.

## Introduction

Coronary artery disease is the most common cause of heart failure worldwide, accounting for 60–70% of cases. [1] For over 30 years, it has been known that left ventricular dysfunction due to coronary artery disease may be reversible in some patients, and that revascularisation can restore contractility. [2] Multiple subsequent follow-up studies have confirmed that in certain patients, revascularisation by coronary artery bypass grafting (CABG) or percutaneous coronary intervention (PCI) can restore left ventricular function, improve ventricular geometry, reduce symptoms and increase overall survival [3, 4].

Myocardial revascularisation in patients with heart failure is challenging. The patient population is elderly, frail and has extensive comorbidities. [5] Patients with heart failure typically have long-standing, complex, multivessel coronary artery disease. Given the challenges of the patient population and coronary anatomy, the procedural risk of revascularisation can be significant. For example, in patients undergoing CABG, operative mortality is more than double if there is severe left ventricular dysfunction. [6] Procedural risks are lower with revascularisation by PCI, but this benefit may be offset by higher rates of incomplete revascularisation. [7] Identifying which patients with heart failure are likely to benefit symptomatically and prognostically from revascularisation, and the optimum approach to achieve this, is therefore important from a patient perspective and to appropriately direct resources.

Imaging to differentiate viable myocardium from scar has entered routine clinical practice as a strategy to identify which patients will benefit from revascularisation.

However, while it is intuitive that revascularisation of non-viable, scar tissue is unlikely to provide improvement in left ventricular function, the value of selecting and revascularising patients with viable but ‘hibernating’ segments based on cardiac imaging has not been definitively established in a randomised controlled trial (RCT) setting. Herein, we review the evidence base for viability-guided revascularisation and discuss whether this remains the correct approach for patients with ischaemic cardiomyopathy.

## Pathophysiology of hibernating myocardium

The term ‘hibernating myocardium’ describes dysfunctional but viable cardiac segments in patients with impaired left ventricular function. [8] As the energy required for contraction exceeds the energy required for cell survival, cardiomyocytes are proposed to enter a state of functional hibernation and stop contracting, a state which can be reversed when myocardial energy supply is restored. [8] Strictly, ‘hibernating’ should only be used in retrospect—as it applies to segments which regain contractility following revascularisation—but in practice is frequently used to describe viable myocardium prior to revascularisation [9].

Hibernation was initially thought to occur due to chronic ischaemia in regions of myocardium with a fixed reduction in blood flow. [10] However, studies of myocardial perfusion using positron emission tomography (PET) showed that myocardial blood flow in hibernating segments was normal, or near normal, in patients with left ventricular dysfunction due to coronary disease.[11, 12] Even where myocardial blood flow was found to be reduced, the magnitude of decrease was insufficient to explain the severity of contractile dysfunction. [13] Myocardial hibernation is therefore now thought of as a form of chronic stunning. This concept has been extrapolated from animal models of acute ischaemia, where transient coronary occlusion leads to acute but reversible contractile dysfunction. [14] In pigs, gradual onset of myocardial ischaemia induces contractile dysfunction without leading to infarction or reduced myocardial perfusion. [15] Episodes of myocardial demand due to arousal or exercise then lead to acute ‘demand-induced ischaemia’ and repeated episodes of acute myocardial stunning.[15, 16] Multiple episodes of acute stunning are proposed to induce chronic, stably depressed left ventricular function. This has been described as the ‘smart heart’ response, an adaptive model to reduce energy requirement and protect against cardiomyocyte cell death [10].

An alternative possibility is that myocardial hibernation is a failed, or stalled, attempt by cardiomyocytes to proliferate. Histologically, hibernating myocardium is characterised by cardiomyocyte dedifferentiation, with loss of sarcomeres, sarcoplasmic reticulum and T-tubules, alongside abundant glycogen and nuclear changes with heterochromatin redistribution. [17] In animal models of regeneration, cardiomyocyte dedifferentiation (with sarcomere disassembly) occurs as the first stage of proliferation and is triggered by hypoxia. [18, 19] In humans, proliferation may stall due to persistent hypoxia or limited energetic substrate. [20, 21] Following revascularisation, cardiomyocyte re-differentiation and proliferation has been observed, which may underlie functional recovery [22].

## Imaging for myocardial viability

Viable but hibernating myocardium is characterised on imaging by preserved cell membrane integrity and metabolism, contractile reserve during stimulation and absence of scar. In contrast, absence of contractile reserve, identification of scar, or loss of membrane integrity or metabolic activity, can be used to define non-viable tissue. Membrane integrity is assessed by uptake of thallium-201 or 99-m technetium tracers, metabolism by uptake of ^18^F–fluorodeoxyglucose (FDG), presence of contractile reserve by stress imaging, e.g. dobutamine stress echocardiography (DSE) or stress cardiac magnetic resonance (CMR) imaging, and presence of scar by late gadolinium enhancement on CMR. Multiple complimentary imaging modalities can therefore be used to identify viable myocardium. Although these vary in reported sensitivity and specificity, with nuclear modalities having greater sensitivity, and wall motion imaging greater specificity, the choice of imaging modality is often primarily influenced by local availability and expertise. A detailed comparison of imaging techniques for assessment of viability is extensively reviewed elsewhere [23, 24].

## Viability testing in clinical practice: observational series

Multiple observational series support the concept that heart failure patients with viable myocardium detected on imaging benefit from myocardial revascularisation. In a 2002 meta-analysis of 24 observational studies, outcomes from 3088 patients who had undergone thallium perfusion imaging, FDG-PET metabolic imaging or DSE, with or without subsequent revascularisation were pooled. [3] In patients with viability, revascularisation was associated with a 79.6% relative reduction in annual mortality compared with medical treatment (16 vs. 3.2%, *p* < 0.0001). In contrast, in patients without viability, there was no statistically significant benefit associated with revascularisation (7.7 vs. 6.2%, *p* = NS).

**Fig. 1:**
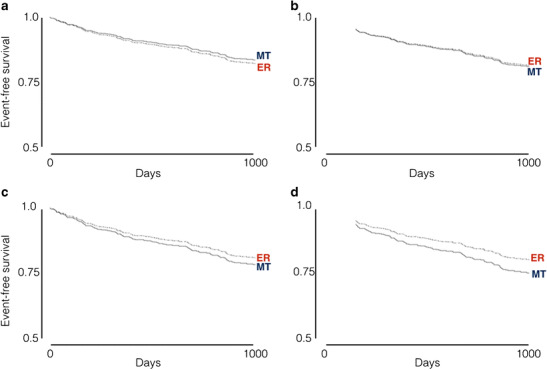
Adjusted Kaplan-Meier survival analysis of all-cause death with early revascularisation (ER) versus medical therapy (MT), according to the percentage of hibernating myocardium: 5% (**a**), 10% (**b**), 15% (**c**) and 20% (**d**). Equipoise between MT and ER is reached at 10% of hibernating myocardium. With greater levels of hibernating myocardium, there is a survival benefit associated with ER. Reproduced from Ling et al. with permission [26]

Observational data from more contemporary series also support the use of viability testing. [25, 26] A 2013 study from the Cleveland Clinic reported outcomes for 648 consecutive ischaemic cardiomyopathy patients who underwent perfusion and metabolic imaging by PET. [26] Patients had a mean ejection fraction of 31%, and early revascularisation (within 92 days) was performed in 199 patients (33%). Patients were followed up for an average of 2.8 years for a primary endpoint of all-cause mortality. Given the non-randomised allocation to revascularisation or medical therapy, propensity analysis was used to adjust for potential confounders. The study identified a significant interaction between revascularisation, percentage of hibernating myocardium, diabetes and outcome (*p* < 0.0009). In medically treated patients, the risk of death increased alongside the percentage of hibernating myocardium, but this was offset in those undergoing revascularisation. Equipoise between medical therapy and revascularisation was present in patients at a threshold of 10% hibernating myocardium, with a progressively greater benefit from revascularisation seen as the proportion of hibernating myocardium increased (Fig. 1).This benefit was only seen in patients without diabetes mellitus, however.

These studies have been used to justify widespread use of viability testing in clinical practice, but remain subject to the uncertainties inherent to a non-randomised design. Specifically, given the lack of blinding to viability test results, it is likely that the decision to undertake revascularisation was strongly influenced by the results of the viability test, introducing bias which is difficult to account for. Furthermore, it is impossible to ensure adequate correction for potential confounders other than viability. Additionally, advances in contemporary heart failure therapy—for example use of ACE inhibition, mineralocorticoid inhibition and device therapy—render extrapolation of historical cohort data to contemporary practice difficult.

## Viability testing in clinical practice: randomised trials

### PARR-2 trial

The PET and Recovery Following Revascularization (PARR-2) study is the only RCT to directly evaluate the role of viability testing on clinical outcomes. [27] PARR-2 enrolled patients with severe left ventricular dysfunction and suspected coronary artery disease being considered for revascularisation or transplantation, and randomised them to FDG-PET imaging (*n* = 218) or standard of care (*n* = 212). The primary endpoint was a composite of cardiac death, MI or recurrent hospital stay. At 1 year, the cumulative proportion of patients who had experienced the primary endpoint was 30% (FDG-PET arm) vs 36% (standard of care arm) (RR 0.82, 95% confidence interval 0.59 to 1.14, *p* = 0.16), suggesting no benefit from FDG-PET imaging.

There are important caveats to the headline result of PARR-2. Importantly, a significant number of patients (30/207, 14.5%) with high or moderate viability did not undergo revascularisation, which could have contributed to the apparent lack of benefit associated with FDG-PET. In a pre-specified subgroup analysis, the authors found that in patients where there was adherence to the PET-guided strategy (76% of patients), this was associated with improved outcome (HR = 0.62 95% CI 0.42 to 0.93; *p* = 0.019). These findings were recently confirmed at 5-year follow-up: there was no significant difference between study arms overall, but a significant reduction in the primary endpoint in the subset in whom PET-guided therapy was followed (HR 0.73, 95% CI 0.54 to 0.99, *p* = 0.042). [28] The reasons for non-adherence to PET guidance included patient comorbidities, renal failure, unsuitability of coronary anatomy for revascularisation and stabilisation of symptoms.

In another post-hoc analysis from PARR-2, the Ottawa-FIVE substudy, a significant benefit from PET guidance was demonstrated in one experienced heart failure centre with imaging expertise. [29] While acknowledged as only hypothesis-generating, it highlights the importance of local expertise, not just in imaging, but in patient selection, revascularisation and downstream heart failure care for this patient population.

### STICH trial

The Surgical Treatment for Ischemic Heart Failure (STICH) study was designed primarily to investigate the role of CABG for revascularisation (and separately, surgical ventricular reconstruction) in patients with severe left ventricular dysfunction. [30] It has also had important implications for viability testing. The revascularisation component of STICH enrolled patients with an ejection fraction of ≤ 35% and randomised them to either CABG with optimal medical therapy (OMT), including ACEI, beta-blockers, statins and diuretics, or OMT alone. The primary endpoint for the trial was death from any cause. Between 2002 and 2007, 1212 patients were enrolled in 99 centres, with 610 patients undergoing CABG plus OMT and 602 patients receiving OMT alone.

The primary outcome of all-cause mortality occurred in 36% in the CABG group compared to 41% in the OMT alone group (HR 0.86, 95% CI 0.72 to 1.04), with the difference failing to reach statistical significance (*p* = 0.12). However, CABG was found to be protective against cardiovascular death (28 vs 33%, HR 0.81, 95% CI 0.66 to 1.00, *p* = 0.05), and protective against the composite of cardiovascular death and hospitalisation (68% OMT vs 58% CABG, HR 0.74, 95% CI 0.64 to 0.85, *p* < 0.001). Aspects of the trial design have been criticised, including the high crossover rates: of 602 patients assigned to OMT, 17% crossed over to CABG before the end of the follow-up period. In a per protocol analysis to exclude crossover, CABG was found to be protective against all-cause mortality (HR 0.76, 95% CI, 0.62 to 0.92, *p* = 0.005). The failure to hit the primary endpoint may also be explained by early mortality in the CABG group associated with the surgical procedure.

The role of viability testing was reported in a dedicated substudy of STICH. [31] Of the 1212 patients enrolled in the revascularisation study, a subset of 601 patients underwent myocardial viability testing. [31] The original trial protocol had intended viability testing to be performed on all patients, but this proved to be a barrier to recruitment and the protocol was revised to make viability testing optional (at the discretion of the recruiting investigator) and to allow use of either SPECT or DSE. The STICH viability substudy reported that on univariate analysis, patients with viable myocardium had a reduced overall rate of death compared to those without (hazard ratio 0.64; 95% confidence interval 0.48 to 0.86; *p* = 0.003). After adjustment in a multivariate model, however, there was a trend towards lower incidence of mortality plus cardiovascular hospitalisation, but this was not statistically significant (55.2% CABG vs 72.1% OMT, HR 0.55, 95% CI 0.55 to 0.85, *p* = 0.3903).

The immediate conclusion from this analysis, that viability does not predict outcome following revascularisation, has been extensively criticised. First, there may have been a lack of power to show a benefit of viability testing, given the relatively small numbers of patients *without* viability in the cohort (19%). Second, the viability group differed to the main trial population with respect to baseline characteristics, drug use and previous PCI, and was therefore felt not to be representative of the ischaemic cardiomyopathy population at large. Third, a high proportion of patients undergoing viability testing were low risk with simple coronary disease (one vessel disease in 25.3%), suggesting that the study may have recruited non-ischaemic cardiomyopathy patients with incidental, bystander, coronary disease, who would not be expected to benefit from revascularisation. Finally, the assessment of viability relied extensively on SPECT and DSE, which was not standardised, and which have been increasingly replaced by PET and CMR in contemporary practice.

Further research is required to clarify the role of viability testing in revascularisation of patients with heart failure. The Alternative Imaging Modalities in Ischemic Heart Failure study (AIMI-HF) aims to compare the effect of heart failure imaging strategies on the composite clinical endpoint of cardiac death, MI, resuscitated cardiac arrest and cardiac hospitalisation. [32] This study is recruiting patients with new or worsening heart failure: left ventricular dysfunction (ejection fraction ≤45% and NYHA class II–IV; or EF ≤30% and NYHA class I) who are then randomised to SPECT or PET/CMR imaging. The study will define ischaemia, viability and scar, and assess the utility of conventional and advanced imaging approaches to alter clinical management and influence hard clinical endpoints.

## Viability-guided revascularisation in practice

Current international guidelines reflect the uncertainty about the role of viability testing. The American College of Cardiology Foundation/American Heart Association have given myocardial viability testing a class IIa (level of evidence B) recommendation, stating ‘viability assessment is reasonable before revascularisation in heart failure patients with coronary artery disease.’ [33] The European Society of Cardiology guidelines only support viability testing (alongside ischaemia testing) at class IIb (level of evidence B), recommending that ‘non-invasive stress imaging’ (CMR, stress echocardiography, SPECT, PET) may be considered for the assessment of myocardial ischaemia and viability in patients with heart failure and coronary artery disease (considered suitable for coronary revascularisation) before the decision on revascularisation [34].

**Fig. 2:**
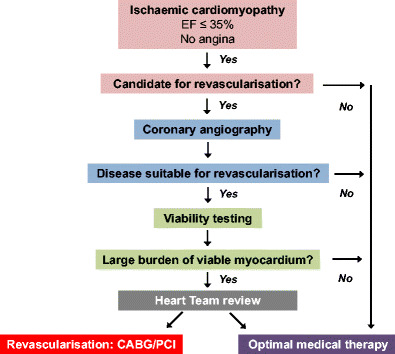
Flow chart for use of viability testing in patients with ischaemic cardiomyopathy. In patients fit for revascularisation, up-to-date assessment of coronary anatomy should be undertaken by coronary angiography. Where revascularisation can be undertaken, viability imaging should be performed. In patients with a large burden of hibernating myocardium, short-term procedural risk may be justifiable to improve long-term outcome. The optimal mode of revascularisation should be assessed by a heart team. Modified from Perera & Redwood with permission [40]

In practice, viability testing does have a role in the work-up of patients with ischaemic cardiomyopathy, but the failure to demonstrate clear benefit of either viability testing or revascularisation itself emphasises the importance of careful patient selection and interpretation. In patients with severe left ventricular dysfunction (i.e. ejection fraction ≤ 35%) and no evidence of angina, the first question is whether the patient is a candidate for revascularisation (Fig. 2). This decision is initially influenced by patient factors, including symptoms, biological age, comorbidities, frailty and quality of life. The next stage is coronary work-up: assessment of the extent and distribution of coronary disease by coronary angiography, underlying ventricular function and assessment for valve disease. In patients in whom there is multivessel coronary disease amenable to revascularisation, we then advise viability testing. If imaging by one modality is inconclusive, a second may be required. Identification of a large burden of hibernating myocardium provides justification to undertake up-front procedural risk and proceed to revascularisation. Whether revascularisation is best achieved by PCI or CABG is dependent on patient factors, preference, coronary anatomy and the likely completeness of revascularisation, and should be discussed in a Heart Team approach.

## PCI versus CABG for revascularisation of ischaemic cardiomyopathy

There are limited data on optimal revascularisation strategy for patients with heart failure. While multiple studies have compared PCI with CABG for patients with coronary artery disease—for example BARI, ARTS, FREEDOM and SYNTAX—these excluded patients with heart failure or severely impaired left ventricular function. In current guidelines, the 2014 ESC/EACTS guidelines give CABG a class I recommendation for revascularisation of patients with chronic heart failure, but given limited data for PCI, it has only a class IIb recommendation. [35] The US, ACCF/AHA guidelines give CABG a class IIb recommendation, with no recommendation for PCI [36].

In a recent meta-analysis of 21 studies (including 3 RCTs) of patients with coronary disease and a left ventricular ejection fraction ≤ 40%, revascularisation by CABG or PCI was compared with OMT. [37] Revascularisation was associated with a significant reduction in mortality compared to OMT (CABG HR, 0.66; 95% confidence interval, 0.61–0.72; *p* < 0.001; PCI HR 0.73; 95% confidence interval, 0.62–0.85; *p* < 0.001). When outcomes from CABG were compared to PCI, CABG was shown to be associated with a further improvement in survival (HR 0.82, 95% CI 0.75–0.90, *p* < 0.001). However, the majority of studies in this meta-analysis were non-randomised and the CABG and PCI populations are therefore likely to be significantly different. In addition, studies from1983 onwards were included, a time period in which there have been significant advances in technique and outcomes from PCI.

In a more recent 2016 registry study, propensity score matching was used to identify 2126 patients with multivessel coronary disease and an ejection fraction ≤ 35% who underwent either PCI or CABG in New York State. [38] At a median follow-up of 2.9 years, PCI was associated with a similar risk of death to CABG (HR = 1.01; 95% confidence interval 0.81–1.28; *p* = 0.91). [38] PCI was associated with a lower risk of short-term stroke, but an increased risk of repeat revascularisation and MI in those with incomplete revascularisation.

Taken together, the available data suggest that if complete revascularisation can be achieved by PCI, this is reasonable and potentially preferable in terms of short-term risk. RCT evidence to support PCI for revascularisation of heart failure patients with viability is anticipated from the ongoing REVIVED-BCIS2 study. [39] This study is recruiting patients with an ejection fraction of ≤ 35%, severe coronary disease (BCIS-1 jeopardy score ≥ 6) and viability in at least four dysfunctional segments (as assessed by DSE or CMR) amenable to revascularisation by PCI. Patients are randomised to either PCI with OMT, or OMT alone. The study primary endpoint is all-cause death or heart failure hospitalisation. As of August 2017, 345 patients of a target 700 had been recruited.

## Conclusions

Viability testing to guide myocardial revascularisation has a place in clinical practice, but at present lacks supporting randomised trial level evidence. This is largely explained by early over-reliance on observational data, leading to few trials being performed. While PARR-2 missed its primary endpoint, it suggested that judicious use of viability testing in patients where revascularisation can be achieved is a useful approach. Selection of ischaemic cardiomyopathy patients with a large burden of viable myocardium, as opposed to scar, is therefore one of multiple factors which should contribute to the complex decision-making process about who to revascularise. Ongoing RCTs in the field, in particular AIMI-HF and REVIVED-BCIS2, should provide important answers with regard to viability imaging and PCI for revascularisation, respectively.
